# Early Prediction of Tacrolimus-Induced Tubular Toxicity in Pediatric Refractory Nephrotic Syndrome Using Machine Learning

**DOI:** 10.3389/fphar.2021.638724

**Published:** 2021-08-27

**Authors:** Xiaolan Mo, Xiujuan Chen, Chifong Ieong, Xia Gao, Yingjie Li, Xin Liao, Huabin Yang, Huiyi Li, Fan He, Yanling He, Yilu Chen, Huiying Liang, Min Huang, Jiali Li

**Affiliations:** ^1^ Department of Pharmacy, Guangzhou Women and Children’s Medical Center, Guangzhou Medical University, Guangzhou, China; ^2^ Institute of Clinical Pharmacology, School of Pharmaceutical Sciences, Sun Yat-sen University, Guangzhou, China; ^3^ Institute of Pediatrics, Guangzhou Women and Children’s Medical Center, Guangzhou Medical University, Guangzhou, China; ^4^ Division of Nephrology, Guangzhou Women and Children’s Medical Center, Guangzhou Medical University, Guangzhou, China; ^5^ Department of Pharmacy, Guangzhou Institute of Dermatology, Guangzhou, China

**Keywords:** tacrolimus, refractory nephrotic syndrome, machine learning, nephrotoxicity prediction models, gene polymorphisms

## Abstract

**Background and Aims:** Tacrolimus(TAC)-induced nephrotoxicity, which has a large individual variation, may lead to treatment failure or even the end-stage renal disease. However, there is still a lack of effective models for the early prediction of TAC-induced nephrotoxicity, especially in nephrotic syndrome(NS). We aimed to develop and validate a predictive model of TAC-induced tubular toxicity in children with NS using machine learning based on comprehensive clinical and genetic variables.

**Materials and Methods:** A retrospective cohort of 218 children with NS admitted between June 2013 and December 2018 was used to establish the models, and 11 children were prospectively enrolled for external validation. We screened 47 clinical features and 244 genetic variables. The changes in urine N- acetyl- β-D- glucosaminidase(NAG) levels before and after administration was used as an indicator of renal tubular toxicity.

**Results:** Five machine learning algorithms, including extreme gradient boosting (XGBoost), gradient boosting decision tree (GBDT), extremely random trees (ET), random forest (RF), and logistic regression (LR) were used for model generation and validation. Four genetic variables, including *TRPC6* rs3824934_GG, HSD11B1 rs846910_AG, MAP2K6 rs17823202_GG, and SCARB2 rs6823680_CC were incorporated into the final model. The XGBoost model has the best performance: sensitivity 75%, specificity 77.8%, accuracy 77.3%, and AUC 78.9%.

**Conclusion:** A pre-administration model with good performance for predicting TAC-induced nephrotoxicity in NS was developed and validated using machine learning based on genetic factors. Physicians can estimate the possibility of nephrotoxicity in NS patients using this simple and accurate model to optimize treatment regimen before administration or to intervene in time after administration to avoid kidney damage.

## Introduction

Tacrolimus (TAC) is the preferred medication for children with refractory nephrotic syndrome (NS). However, the clinical use of TAC is limited by its adverse effects, especially nephrotoxicity. Renal interstitial fibrosis and tubular atrophy are the main manifestations of nephrotoxicity, and the incidence of nephrotoxicity has a large individual difference (about 4.7∼20.0%) (Choudhry et al., 2009; Roberti and Vyas, 2010; Gulati et al., 2012; Jahan et al., 2015). If nephrotoxicity occurs during TAC treatment, the reduction or withdrawal of TAC is needed, which may result in treatment failure. Furthermore, damage of kidney function can lead to end-stage kidney disease even disability and death. Therefore, the early prediction of TAC-induced nephrotoxicity is conducive to the selection of appropriate regimens and interventions to ensure efficacy and avoid side effects. Thus, it is very necessary to establish a pre-administration prediction model of TAC-induced tubular toxicity and to excavate the relevant factors that can accurately predict tubular toxicity.

Tacrolimus, an important calcineurin inhibitor (CNI), is widely used in kidney transplantation and NS. However, there are only few reports focused on the risk factors of TAC-induced nephrotoxicity in NS (Morgan et al., 2011; Sinha et al., 2013; Gao et al., 2020). The risk factors excavated by these models include TAC trough concentration, diarrhea, proteinuria duration, high blood pressure, etc. However, these studies have certain limitations: First, these risk-factor models were generated using traditional logistic regression (LR), which may not be the optimal method because of its low prediction performance; Second, Gao et al. (2020) used serum creatinine as the indicator of nephrotoxicity , which may not sensitively reflect kidney damage. Studies have shown that a significant increase in serum creatinine can only be seen when kidney function drops to 50% of normal levels (Price, 1992). Therefore, the model based on this insensitive indicator may not be able to identify patients with mild to moderate nephrotoxicity. In addition, serum creatinine reflects glomerular damage instead of renal tubular damage. Therefore, it could not be used as an accurate marker of tubular toxicity of TAC. Third, the existing studies only investigated clinical variables such as gender, age, and the pathological classification of NS. Although factors such as age and weight have been shown to affect the pharmacokinetics of TAC in organ transplantation and NS, they could not fully explain the individual variations in TAC-induced nephrotoxicity.

Studies have shown that 20–95% of individual differences in drug pharmacokinetics-pharmacodynamics (PK-PD) are caused by genetic factors especially single nucleotide polymorphisms (SNPs) (Evans and Mcleod, 2003). For example, from the PK perspective, TAC is a substrate of CYP3A4/5 and P-gp. Current studies in organ transplantation and NS have consistently shown that CYP3A5*3 significantly affects the pharmacokinetics of TAC, the TAC concentration of *3 carriers is higher than that of non-carriers (Zhang et al., 2005; Renders et al., 2007; Wang D. et al., 2019; Wang X. et al., 2019). Additionally, some transcriptional regulators such as NF-κB (encoded by NFKB1 and RELA), inflammatory cytokines such as IL2 (encoded by IL2) and IL10 (encoded by IL10) can regulate the expression and activity of CYP3A and P-gp (Abdel-Razzak et al., 1993; Stein et al., 1996; Tinel et al., 1999; Gu et al., 2006). Therefore, the corresponding gene polymorphisms may affect the pharmacokinetic of TAC. Prednisone, which is often combined with TAC for treating NS, can induce CYP3A (Pichard et al., 1992). While prednisolone is a weak inhibitor of CYP3A4 (Lam et al., 2008). Hormone convertase 11β-HSD1 (encoded by HSD11D1) is a metabolic enzyme that converts prednisone to prednisolone, which gene polymorphism may also affect the TAC pharmacokinetic. As for PD, cytokines such as IL2, IL2-Rα, and IL-13, and proteins such as MAPK and TGFβ constitute the PD pathway of TAC. Therefore, the mutations of the above relevant genes may cause large individual differences in TAC PK and PD (Hoyos et al., 1989; Cardenas et al., 1994; Macián et al., 2000; Jeffrey et al., 2007). For example, the CNI-induced nephrotoxicity (tubular interstitial fibrosis) is correlated with the overexpression of TGFβ protein (Border and Noble, 1994; Khanna et al., 2002; Wolf, 2006). Besides, the leakage of blood protein from the urine is an important characteristic of NS. Therefore, TAC will leak into the urine with binding protein because of its high protein binding rate. Some studies found that the gene polymorphisms of podocyte proteins related to renal filtration can significantly affect the concentration and efficacy of TAC in NS children (Mishra et al., 2014; Mo et al., 2020). which may also affect the nephrotoxicity. Thus, the gene polymorphisms of proteins related to TAC PK-PD pathways, kidney podocytes, transcriptional regulators, inflammatory cytokines, and hormone convertases may be the important reasons for the individual differences in TAC-induced nephrotoxicity. These genetic factors may fully explain the nephrotoxicity together with clinical variables.

Therefore, an early and accurate model with comprehensive clinical and genetic variables is needed to predict TAC-induced nephrotoxicity in NS children. In recent years, thanks to the powerful data mining and computing capacity, machine learning has gradually played an important role in the biomedical field, especially in the classification and regression of disease diagnosis, treatment, and prognosis. Tang et al. (2017) established a steady-state dose prediction model of TAC in the kidney transplant population using multiple machine learning algorithms, with a prediction accuracy over 70%. Lin et al. (2015) used machine learning to develop an automatic recognition system for methotrexate hepatotoxicity. These models might provide convenient, scientific, and accurate clinical decision-making in diagnosis, treatment, and toxicity in the future. Nonetheless, there have not been any TAC-induced nephrotoxicity prediction models in NS generated by machine learning so far.

Therefore, this study aims to develop an early, sensitive, and accurate TAC-induced nephrotoxicity prediction model based on clinical and TAC-related genetic variables, which is conducive to the doctors’ treatment decisions.

## Methods

### Study Design and Population

We performed two observational cohort studies at the Guangzhou Women and Children’s Medical Center. The derivation cohort, which consisting of retrospectively collected data from records of children who visited the department of nephrology and were treated with TAC between June 2013 and December 2018, was used to generate the prediction model. The validation cohort, which consisting of prospectively collected data from records of children who were treated with TAC between January 2019 and October 2019, was used for the external validation of the model. The inclusion criteria are: 1) Met the diagnosis criteria of refractory NS, including steroid-dependent NS, steroid-resistant NS, and frequently relapsing NS (Lombel et al., 2013). 2) Age ≤16 years old. 3) Patients regularly received TAC and low-dose corticosteroids for more than 1 month, and the trough concentration ≤12 ng/ ml during follow-up. The exclusion criteria are: 1) Steroid-sensitive NS, secondary NS, hereditary NS, etc. 2) Co-treatment with drugs that may cause nephrotoxicity within 2 months prior to the onset of nephrotoxicity, such as mycophenolate mofetil, high-dose methylprednisolone, cyclophosphamide, diuretics, contrast agents, biological agents, first or second generation of cephalosporin, vancomycin, etc. 3) Patients with impaired liver/kidney function or other malignant diseases (such as cancer). 4) Patients had elevated NAG levels at baseline before they were given TAC. 5) Patients with poor compliance. A total of 274 NS children treated with TAC were screened out, but 218 patients were eventually included for modeling and validating. Furthermore, we continued to collect and follow up 35 NS patients from January 2019 to October 2019 for external validation. Because of loss of follow-up, abnormal baseline NAG levels before administration, undetected key genes, and the combination of contrast agents and other interfering agents, 24 patients were excluded during the follow-up, 11 patients were eventually included in the validation.

This study was approved by the Ethics Committee of our hospital (No. 201509), and also registered on Clinical Trial (NCT02602873). All patients’ guardians signed the informed consent before the trial. Besides, the data used in this study were anonymous.

### Assessment of Tubular Toxicity

All patients received a double immunosuppressive regimen consisting of tacrolimus (Prograft™, Astellas, Killorglin, Ireland) and low-dose prednisone or methylprednisolone (Guangdong Huanan Pharmacy Ltd., Dongguan, China; Pfizer Italia S. r.l., Italy). The initial dose of tacrolimus (0.10–0.15 mg/ kg twice daily) was given to patients, and doses were subsequently adjusted to achieve a target trough concentration (C_0_) of 5–10 ng/ ml.

Renal biopsy, the gold standard for nephrotoxicity assessment, can evaluate the kidney pathological changes. However, most patients do not accept it since it is an invasive test. Thus, ethical issues limited the number of participants. Moreover, renal biopsy is a post-nephrotoxicity test, which is difficult to predict the TAC-induced nephrotoxicity early and sensitively because of its hysteresis. Besides, serum creatinine level, which has low sensitivity and accuracy, is also not a good indicator of tubular toxicity. Therefore, we used urine N- acetyl- β-D- glucosaminidase (NAG) as a tubular toxicity indicator. Within 48 months after the initiation of continuous TAC treatment, if NAG level increases above the upper limit of the normal range after excluding other factors (such as combined use of contrast agents, heavy proteinuria, abnormal baseline NAG level, etc.), early TAC-induced tubular toxicity is defined according to the criteria of drug side effects (Naranjo et al., 1981). NAG is a lysosomal enzyme derived from proximal tubular epithelial cells. This enzyme is usually not filtered through the glomerulus, thus the excretion of this enzyme in the urine is not affected by the same enzyme in the blood, which can specifically reflect the trauma of renal parenchyma.^8^ In addition, the renal tubule is mainly damaged in the acute or chronic CNI-induced nephrotoxicity, which may cause the rapid induction of lysosomal enzyme release (Naesens et al., 2009), and the NAG activity often increases earliest in the urine (Marchewka et al., 2009). Plus NAG is stable in urine, it is often used as an important and reliable indicator of early CNI-induced nephrotoxicity (Price, 1992; Marchewka et al., 2009).

### Clinical Features, Genetic Variables and Genotyping

Various clinical variables before administration and within 48 consecutive months after administration were collected, including hematological characteristics, urine characteristics, drug information, disease diagnosis, pathological examination, etc. When the NAG level becomes abnormal in urine, we should particularly pay attention right away to the time, age, weight, etc. All the complications and the drugs used in the same period should be recorded in detail, which is the basis for the identification of side effects. All clinical variables used in feature selection and their abbreviations are shown in Supplementary Table S1.

The genetic variables (SNPs) were comprehensively detected, including genes related to TAC PK-PD pathways, hormone convertase, kidney podocytes, transcriptional regulators, inflammatory cytokines, etc. These SNPs may have a significant effect on the concentration and the efficacy of TAC (Hoyos et al., 1989; Pichard et al., 1992; Abdel-Razzak et al., 1993; Border and Noble, 1994; Cardenas et al., 1994; Stein et al., 1996; Tinel et al., 1999; Macián et al., 2000; Khanna et al., 2002; Zhang et al., 2005; Gu et al., 2006; Wolf, 2006; Jeffrey et al., 2007; Renders et al., 2007; Lam et al., 2008; Mishra et al., 2014; Wang D. et al., 2019; Wang X. et al., 2019; Mo et al., 2020). Therefore, they may also affect the TAC-induced nephrotoxicity. The inclusion criteria based on these SNPs are: 1) potentially functional mutant located at exon, 5’-UTR, 3’-UTR, and some intron; 2) affecting the microRNA binding sites activity; 3) the minor allele frequency (MAF) reported in HapMap was ≥5% for Chinese subjects. 2 ml of peripheral blood was collected to extract DNA using Genome TIANGEN Blood DNA Extraction Kit (DP348, Beijing, China). The published PCR-RFLP method Li et al. (2011), Zhang et al. (2013) and MALDI-TOF MS method (Agena Bioscience MassARRAY^®^ system, Agena Bioscience, San Diego, CA, United States) were used for all detection of SNPs. The specific information of all SNPs is shown in Supplementary Table S2. The Hardy-Weinberg equilibrium test was performed using χ2 test or Fisher’s exact test (two-sided). The sample size was calculated by using the PASS software (version 11.0.7; PASS, NCSS, LLC).

### Machine Learning

A single-sample Kolmogorov-Smirnov test of normality was performed for all variables. Data were expressed as the median and range or mean ± SD, depending on the data type.

**FIGURE 1:**
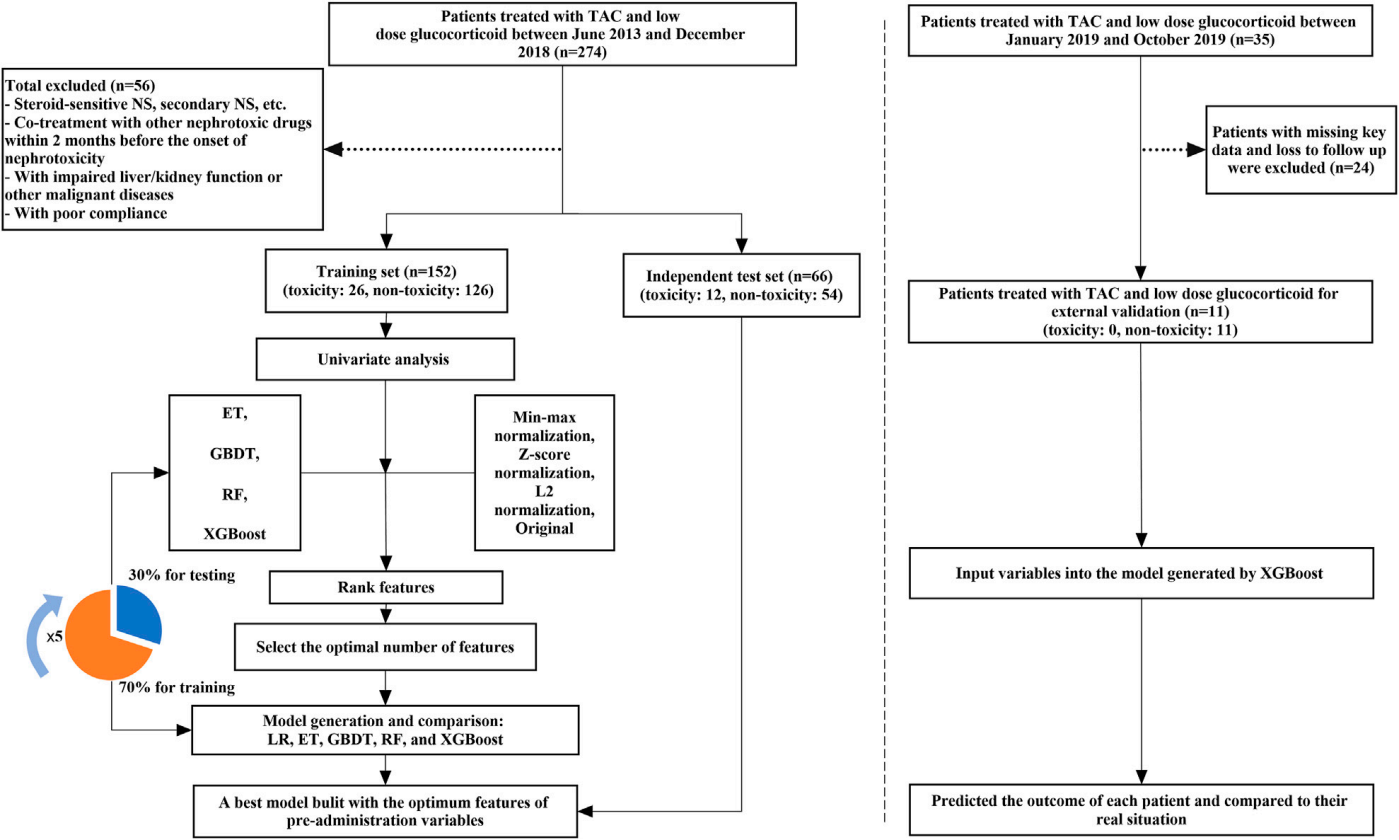
The flowchart of model generation and validation. The left side of the dotted line is the process of model generation, while the right side is the external validation of the model. ET, Extremely Randomized Trees; GBDT, Gradient Boosting Decision Tree; LR, Logistic Regression; RF, Random Forest; XGBoost, eXtreme Gradient Boosting.

Based on the clinical and genetic variables, we used machine learning algorithms to generate a TAC-induced nephrotoxicity prediction model in NS children. Machine learning can be divided into 3 steps: 1) Data pre-processing 2) Feature selection 3) Model generation and validation. Besides, five-fold cross-validation was used to evaluate the performance and prediction error of feature selection and model generation. The flowchart of machine learning is shown in Figure 1. Machine learning techniques were implemented in Python 3.6.5 using Scikit-learn 0.19.1. GraphPad Prism 5 and CorelDRAW X7 were used for graphing. *p* values of <0.05 were considered significant statistically.

### Data Preprocessing

Variables with missing rate >10% were removed. The missing values of continuous and categorical variables were filled with the mean and mode values, respectively. The continuous variables were normalized by z-score normalization. While the categorical variables were transformed into dummy variables.

### Feature Selection

Univariate analysis was used to explore the correlation between each variable and TAC-induced nephrotoxicity (NAG). The negative variables (*p* > 0.05) were excluded. Then, data transformation was carried out on the original continuous variables to form the other two kinds of data: min-max normalization and L2 normalization. Four machine learning algorithms, extreme gradient boosting (XGBoost), gradient boosting decision tree (GBDT), extremely random trees (ET), and random forest (RF) combined with SMOTE were used to analyze the above four forms (z-score normalization, min-max normalization, L2 normalization, and original) of data and generate models. The important contribution of each variable in the four models was evaluated by the median important ranking. Then starting from the variable with the highest contribution, XGBoost algorithm combined with the five-fold cross-validation method iteratively generated a new model by adding one variable at a time. Then the Accuracy, Sensitivity, and AUC results of the model were evaluated until the last variable is added. The optimal variables which constitute the final feature set were determined by the above three indicators.

### Model Generation and Validation

The LR, ET, GBDT, RF, and XGBoost algorithms were used to analyze the above final feature set and generate nephrotoxicity prediction models. During model generation, 218 NS patients were randomly divided into a training set and a test set according to the ratio of 7:3. Moreover, the five-fold cross-validation method was used to generate models based on the training set.

We also additionally collected 35 patients for further external validation and 11 patients were finally included.

## Results

### Patients Characteristics

A total of 218 children with refractory NS were collected in this study, including 159 males and 59 females. The average age of the children was 5.6 ± 3.4 years old, and the average pre-administration weight was 19.9 ± 7.9 kg. According to the evaluation criterion of drug adverse effects, 38 patients have TAC-induced nephrotoxicity, while 180 patients did not. The incidence of nephrotoxicity was 17.4 %. Besides, we collected a total of 47 pre-administration clinical variables and 244 genetic variables. Table 1 describes the baseline characteristics of our study population. The genotypes eventually included in this study all meet the Hardy-Weinberg equilibrium.

**TABLE 1:** Demographics and main clinical characteristics of all pediatric patients with refractory nephrotic syndrome.

Characteristics	Values (*n* = 218)
Male/Female	159/59
Age (years)	5.6 ± 3.4
Weight (kg)	19.9 ± 7.9
Alanine transaminase (ALT, U/L)	19.157 ± 13.631
Aspartate transaminase (AST, U/L)	26.609 ± 10.758
Serum creatinine (SCr, µmol/L)	32.329 ± 34.011
Blood TAC concentration (ng/ml)	6.110(1.980∼20.800)

Data are presented as median with range, mean ± standard deviation or amount.

### Feature Selection

In the univariate analysis, variables that significantly influenced TAC-induced nephrotoxicity (NAG) including pre-administration urinary erythrocytes (URBC0) (*p* = 0.010), *TRPC6* rs3824934_GG (*p* = 0.030), *HSD11B1* rs846910_AG (*p* = 0.045), MAP2K6 rs17823202_GG (*p* = 0.044), *SCARB2* rs6823680_CC (*p* = 0.022), etc.

**FIGURE 2:**
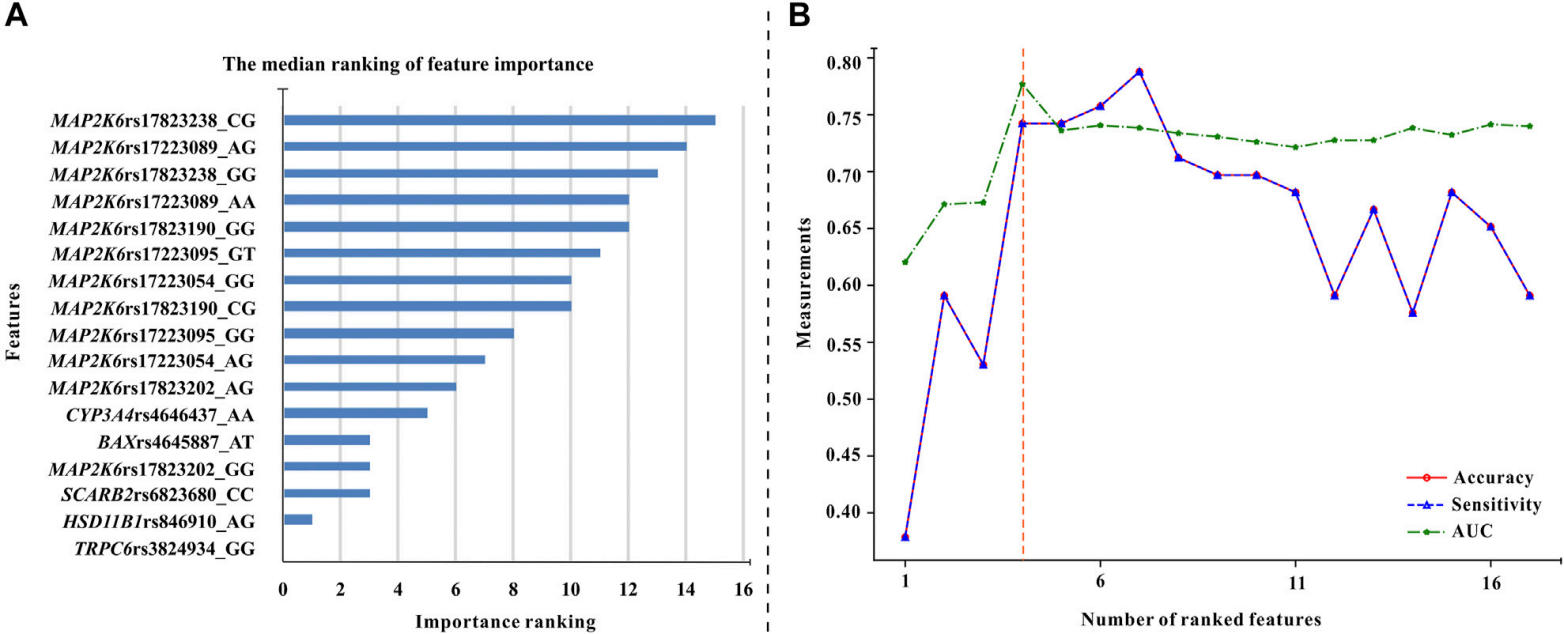
The procedure of feature selection. **(A)** The median values of feature importance ranking (each variable has 16 ranking values). The smaller the median value, the more important the variable **(B)** The overall variation of accuracy, sensitivity, and AUC of the models used for excavating the optimal feature combination. These models were generated by the XGBoost algorithm and five-fold cross-validation. Each variable was added to the models in turn (Starting from the variable with the highest median values until all variables were added to the models). It can be seen that the model built by the first four variables has the best AUC and relatively high accuracy and sensitivity values. Therefore, these four variables (TRPC6 rs3824934_GG, HSD11B1 rs846910_AG, MAP2K6 rs17823202_GG, and SCARB2 rs6823680_CC genotypes) are the optimal feature set.

**FIGURE 3:**
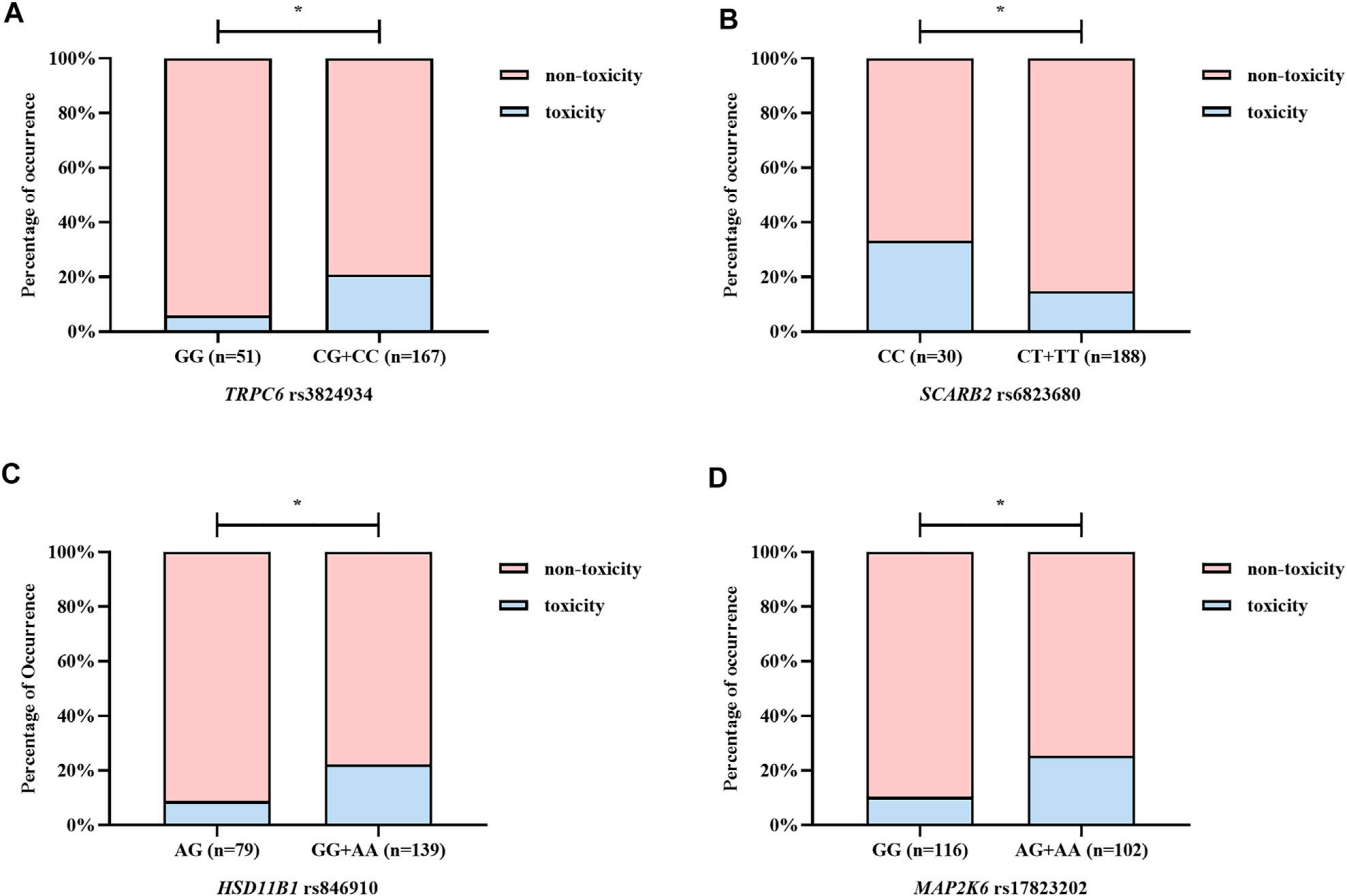
The correlation between the optimal variables and NAG nephrotoxicity. **(A–D)** illustrate TRPC6 rs3824934, SCARB2 rs6823680, HSD11B1 rs846910, and MAP2K6 rs17823202, respectively. Patients with TRPC6 rs3824934_GG, HSD11B1 rs846910*_*AG, and MAP2K6 rs17823202_GG genotypes have a lower rate of NAG nephrotoxicity, while carriers of SCARB2 rs6823680_CC genotype have higher nephrotoxicity rate. *p < 0.05.

Based on four forms of transformed data, we used ET, GBDT, RF, and XGBoost algorithms to generate models and ranked the median important contribution of variables. The smaller the median value, the more important the variable. Figure 2A shows the median important ranking of each variable. The following genotypes have much more important contributions: *TRPC6* rs3824934_GG, HSD11B1 rs846910_AG, *SCARB2* rs6823680_CC, and so on. Figure 2B shows the process of stepwise forward modeling for the best features set. The model containing *TRPC6* rs3824934_GG, HSD11B1 rs846910_AG, MAP2K6 rs17823202_GG, and SCARB2 rs6823680_CC genotypes has the relatively best accuracy, sensitivity, and AUC results. Therefore, these four variables are the best feature set, which was used to generate and validate the final NAG toxicity prediction model. The correlation between these four genetic variables and the occurrence of nephrotoxicity (NAG) is shown in Figure 3. Patients with TRPC6 rs3824934_GG genotype have less nephrotoxicity than those carried CG + CC (*p* = 0.030); carriers of the AG genotype of HSD11B*1* rs846910 have less nephrotoxicity than patients carried AA + GG (*p* = 0.045); the nephrotoxicity in carriers of MAP2K6 rs17823202_GG is less than the patients with AG + AA genotype (*p* = 0.044); patients with SCARB2 rs6823680_CC genotype have more nephrotoxicity than patients carried CT + TT (*p* = 0.022).

### Model Performance and Comparison

**FIGURE 4:**
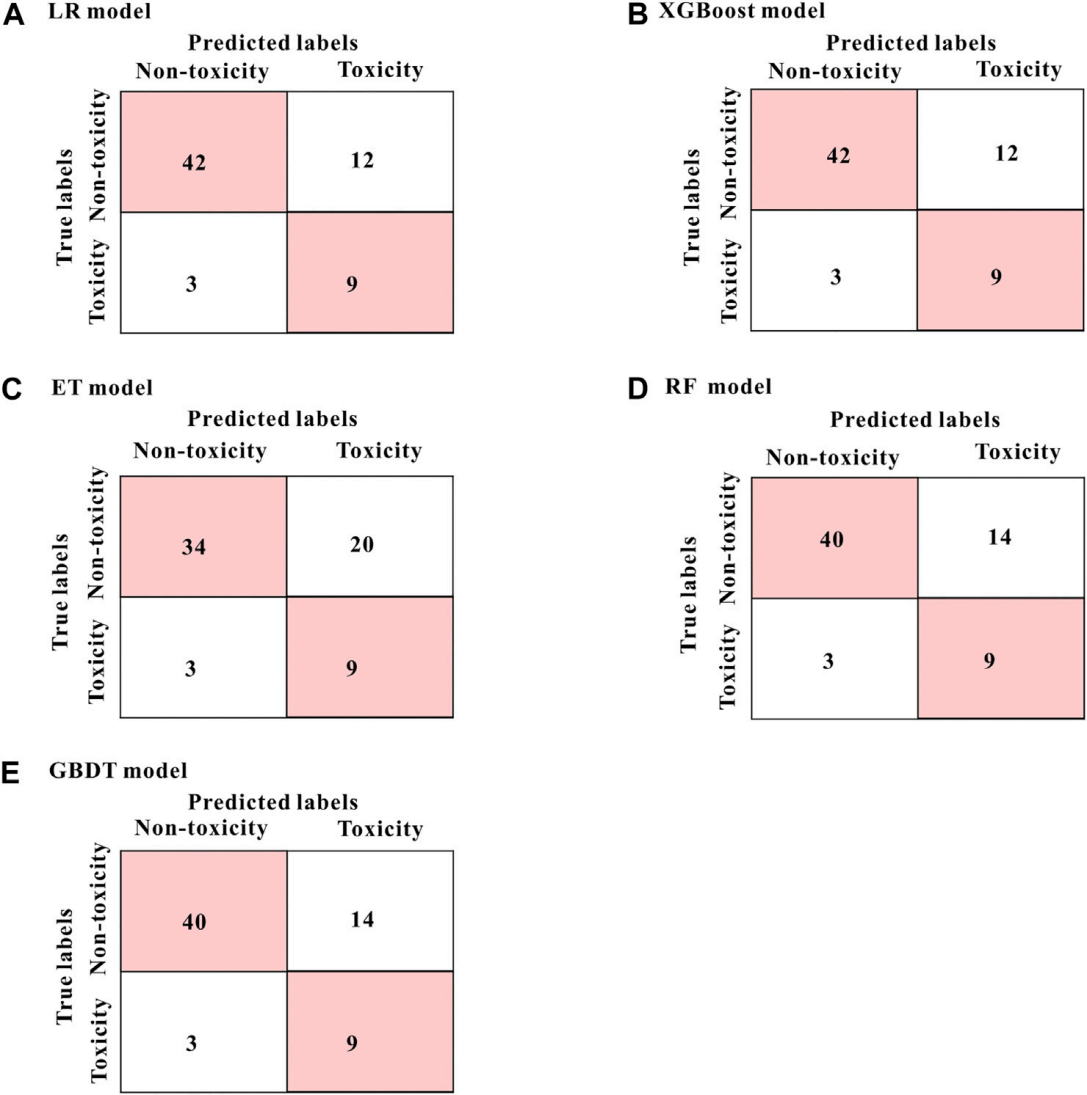
The mixed matrix results of five prediction models in the test set **(A–E)**. For example, in the XGBoost model (shown in **B**), 42 of the 54 true non-toxic patients (pink grids in the upper left corner) were accurately predicted, while 9 of the 12 true toxic patients (pink grids in the lower right corner) were accurately predicted. Other models **(B–E)** are in the same analogy. All results were at the threshold of 0.5.

The performance of the five prediction models based on the above four variables in the test set were as follows: sensitivity 75.0%, specificity 63.0∼77.8%, accuracy 65.2∼77.3%, AUC 74.9∼78.9% (Table 2). The models generated by the LR and the XGBoost algorithm have the best specificity and accuracy results. From the mixed matrix results of the five prediction models, the XGBoost and LR models also show the best accuracy. True non-toxic patients were accurately predicted by 77.8% (42/54), while true toxic patients were accurately predicted by 75.0% (9/12) (Figure 4). However, the XGBoost algorithm has the best AUC result (0.789). Therefore, the XGBoost model has the best prediction performance compared to the other four algorithms. Supplementary Table S3 shows the contribution of each variable to the outcome in the five models. For example, the MAP2K6 rs17823202_GG genotype has the greatest contribution to the XGBoost model.

**TABLE 2:** The performances comparison of five models.

Model	Sensitivity	Specificity	Accuracy	AUC
LR	0.750	0.778	0.773	0.771
XGBoost	0.750	0.778	0.773	0.789
ET	0.750	0.630	0.652	0.749
RF	0.750	0.741	0.742	0.783
GBDT	0.750	0.741	0.742	0.755

### External Verification and Clinical Application

We also additionally collected 35 patients for external validation of the XGBoost model, 11 patients were finally enrolled. The result shows that 9 of 11 patients could be correctly predicted, with an accuracy of 81.8 %. In clinical practice, we input their genetic variables (TRPC6 rs3824934_GG, *HSD11B1* rs846910_AG, MAP2K6 rs17823202_GG, and SCARB2 rs6823680_CC) into the XGBoost model and predicted whether they had nephrotoxicity after TAC treatment (Table 3).

**TABLE 3:** The result of nephrotoxicity after treating with TAC of clinical patient predicted by XGBoost model.

XGBoost model	Patient name	Input variables				Output
TRPC6rs3824934_GG	HSD11B1rs846910_AG	SCARB2rs6823680_CC	MAP2K6rs17823202_GG
	XXX	1	0	0	1	Non-toxicity

The patient named XXX in this table was treated with TAC in clinic practice.

We input his genetic variables into the XGBoost model.

After calculating the data by the model, the output result is no toxicity.

The “output” represents the prediction result of the occurrence of nephrotoxicity.

## Discussion

In this study, we used machine learning algorithms to generate a series of nephrotoxicity prediction models of TAC in NS based on clinical and genetic variables. Four genetic variables, including *TRPC6* rs3824934_GG, HSD11B1 rs846910_AG, MAP2K6 rs17823202_GG, and SCARB2 rs6823680_CC were excavated that can significantly affect the nephrotoxicity of TAC. The model with the above four genetic variables generated by the XGBoost algorithm has the best predictive performance which could accurately identify 78.9% of patients.

To our knowledge, this is the first study using machine learning to generate a TAC-induced nephrotoxicity prediction model in NS, which was based on comprehensive clinical and genetic variables. To date, only few studies focused on the risk factors of TAC-induced nephrotoxicity in NS (Morgan et al., 2011; Sinha et al., 2013; Gao et al., 2020). For example, Gao et al. (2020). established a model using LR to evaluate the risk factors of TAC-induced acute nephrotoxicity. TAC trough concentration and diarrhea were excavated as the risk factors of acute nephrotoxicity, while co-treated with Huaiqihuang granules was the protective factor. But, they used serum creatinine as the indicator of kidney damage, which is less sensitive than our indicator (NAG). Similarly, the other studies also used traditional LR methods to evaluate the effect of clinical variables such as gender, age, and histopathological features on TAC-induced nephrotoxicity (Morgan et al., 2011; Sinha et al., 2013). From the above studies, the variables investigated are not comprehensive enough, and all of them applied the traditional LR method, which leads to the low predictive performance of models.

On the contrary, machine learning often yields models with high predictive performance. Compared with traditional methods, machine learning can handle more complex, high-dimensional, and interactive variables. And the models built by machine learning have higher accuracy and stronger generalization ability (Kruppa et al., 2012; Lee et al., 2018). Among the machine learning algorithms, XGBoost Chen and Guestrin (2016) and GBDT (Tabrizchi et al., 2020) are both boosting ensemble learning algorithms. By continuously adjusting the weight of each sample, multiple different weak classifiers are established, and then combined into a strong classifier to improve algorithm prediction and generalization capabilities. Besides, the XGBoost algorithm introduces a regular term, which can prevent overfitting better than GBDT. RF Shi and Horvath, 2006; Pal (2005) and ET (Geurts et al., 2006) are bagging ensemble learning algorithms. Multiple weak classifiers are established through repeated sampling training data sets with replacement, and then combined into strong classifiers to improve algorithm prediction and generalization capabilities. Profiting from these advantages, machine learning technology has been widely applied in the classification and regression of disease diagnosis, treatment, and prognosis (Lin et al., 2015; Tang et al., 2017; Athreya et al., 2019). For example, we previously used machine learning algorithms to develop an artificial intelligence diagnosis model for pediatric respiratory diseases (Liang et al., 2019). Also, we established a model to predict the efficacy of methotrexate in juvenile idiopathic arthritis using machine learning (Mo et al., 2019). Similarly, besides the traditional LR method, we also used four advanced machine learning algorithms (ET, GBDT, RF, and XGBoost) to generate nephrotoxicity prediction models. From Table 2, we can find that the XGBoost model can accurately predict 78.9% of patients, which is better than the traditional LR model. Like other relevant studies (Lee et al., 2018; Mo et al., 2019), our study also suggested that machine learning methods had better predictive performance than traditional statistical methods.

An accurate prediction model requires not only the advanced modeling method but also mining as comprehensive features as possible. Recent studies have found that genetic polymorphisms related to TAC PK-PD pathways, kidney podocytes, transcriptional regulators, inflammatory cytokines, hormone convertases, etc. can affect the PK or the PD process of TAC (Hoyos et al., 1989; Abdel-Razzak et al., 1993; Border and Noble, 1994; Cardenas et al., 1994; Stein et al., 1996; Tinel et al., 1999; Macián et al., 2000; Zhang et al., 2005; Gu et al., 2006; Jeffrey et al., 2007; Renders et al., 2007; Wang D. et al., 2019; Wang X. et al., 2019). But to our knowledge, there have not been any studies exploring the correlation between gene polymorphisms and TAC-induced nephrotoxicity in NS patients. Here, we first comprehensively examined the effect of the above gene polymorphisms and clinical features on TAC-induced nephrotoxicity in the NS population. Unlike other studies (Morgan et al., 2011; Sinha et al., 2013; Gao et al., 2020), no clinical variables were excavated to have a significant effect on TAC-induced renal tubular toxicity (NAG) in our study. Although the URBC0 significantly affected NAG in the univariate analysis, it was not included in our final model after screening by machine learning algorithms. In addition, other studies used serum creatinine level as a representative for nephrotoxicity, but we used a more sensitive NAG to indicate renal tubular toxicity (Price, 1992; Marchewka et al., 2009). Moreover, these studies have not explored other features such as genetic variables, which may contribute significantly beyond clinical characteristics to nephrotoxicity.

Our results supported this view. The variables included in the final model are all genetic variables, including TRPC6 rs3824934_GG, *HSD11B1* rs846910_AG, MAP2K6 rs17823202_GG, and SCARB2 rs6823680_CC genotypes. From Figure 3 we can find that patients with TRPC6 rs3824934_GG, HSD11B1 rs846910_AG, and MAP2K6 rs17823202_GG genotypes are less likely to have TAC-induced nephrotoxicity (NAG) than non-carriers, while carriers of SCARB2 rs6823680_CC has a higher incidence of nephrotoxicity than non-carriers. TRPC6 is an ion channel protein located on the kidney podocyte membrane. The overexpression of TRPC6 may cause glomerular diseases such as focal segmental glomerular sclerosis (FSGS) and minimal change disease (MCD) (Winn et al., 2005). TAC could downregulate the expression of TPRC by inhibiting the calcineurin, which can alleviate the damage of podocyte (Nijenhuis et al., 2011; Ma et al., 2015). A study has shown that TRPC6 rs3824934 C > G mutation may increase the transcription and expression of TRPC6, which may be implicated in the development of steroid-resistant nephropathy (Kuang et al., 2013). However, we excavated the GG genotype as a protective factor of nephrotoxicity. Our result differs from the above study, perhaps because the two study outcomes are inherently different and difficult to compare directly. Moreover, from our results, it is difficult to fully explain the occurrence of TAC-induced nephrotoxicity with single-site mutation, which should be the result of multiple gene mutations. 11β-hydroxysteroid dehydrogenase, which metabolizes prednisone to active prednisolone, was encoded by HSD11B1. Prednisone can induce CYP3A4 and P-gp, while prednisolone is a weak inhibitor of CYP3A4 (Pichard et al., 1992; Lam et al., 2008). Therefore, the polymorphism of HSD11B1 may affect the PK and PD of TAC. The HSD11B1 rs846910*_*AG genotype was found to be a protective factor of nephrotoxicity in our study. Furthermore, Liu et al. (2015) found that carriers of HSD11B1 rs846910_AA had a lower TAC concentration . We speculated that patients with the A allele may have a relatively low TAC concentration, which could protect against nephrotoxicity. However, more clinical verification is needed. MAP2K6 encodes an upstream protein kinase of the TAC PD pathway (P38 pathway) (Matsuda et al., 2000). The activation of the p38 pathway by MAP2K6 can cause kidney damage (Ma et al., 2007). Renal vasoconstriction is an important manifestation of CNI-induced nephrotoxicity, which might be caused by the inhibition of COX-2 expression (Hocherl et al., 2002; Hocherl et al., 2004). Studies have found that the MAPK kinase in the p38 pathway regulates the stability of COX-2 mRNA (Lasa et al., 2000), which may indirectly influence TAC-induced nephrotoxicity. In our study, carriers of MAP2K6 rs17823202_GG are associated with less nephrotoxicity. We speculated that the mutation of the G allele may inhibit the activation of the renal P38 pathway and protect the kidney function. SCARB2 encodes a type III transmembrane glycoprotein (LIMP-2) which is primarily located in lysosomes and late endosomes. Studies have shown that the lack of SCARB2, which may be related to glomerulosclerosis (Berkovic et al., 2008), can also lead to proteolysis failure resulting in tubular proteinuria (Desmond et al., 2011). Additionally, acute arteriopathy is one of the manifestations of acute CNI-induced nephrotoxicity (Naesens et al., 2009), which may be caused by the activation of the RAS system (Hocherl et al., 2004; Ruster and Wolf, 2006; Lee et al., 2012). The deficiency of LIMP-2 may indirectly activate the RAS system by increasing the level of renin which is an important upstream substance in the RAS system (Lee et al., 2012). Therefore, the LIMP-2 may be correlated to the TAC-induced nephrotoxicity. We found that SCARB2 rs6823680_CC is a risk factor of nephrotoxicity, which may be due to the decreased expression of SCARB2 in patients with CC genotype and thus lead to kidney damage. These polymorphisms discussed above were first excavated that related to TAC-induced nephrotoxicity, but the mechanisms require further validation.

In addition, we also prospectively recruited 11 children with refractory NS for external validation. The above four genetic polymorphisms were detected and input into the XGBoost model to predict TAC-induced nephrotoxicity (Table 3). The outcome of 9 patients was correctly predicted, with an accuracy of 81.8%. Thus, this model has a good ability of extrapolation, which can effectively assist doctors in making treatment decisions before administration. When the predicted outcome is non-toxic, a TAC treatment regimen may be used; on the contrary, alternatives may be chosen; where TAC is necessary in the absence of alternatives, it should be carefully applied under frequently monitoring and timely intervention. This study was limited by the number of NS patients and single center. Besides, we used the NAG level as the indicator of nephrotoxicity, while renal biopsy may be a better choice. Therefore, we hope to enroll more NS patients and perform multi-center research to verify our model.

In summary, we first used advanced machine learning to establish and validate a TAC-induced tubular toxicity prediction model based on comprehensive clinical and genetic variables. Besides, genes encoding kidney podocytes, hormone convertases, and proteins of TAC PD pathways were first screened out. These SNPs are closely related to the TAC PK-PD process. This reminds us these SNPs are essential for fully predicting TAC-induced nephrotoxicity. With this pre-administration model, clinicians can estimate the possibility of TAC-induced nephrotoxicity in NS patients, which is beneficial to the TAC prescription and the timely intervention after administration, to avoid kidney damage.

## Data Availability

The original contributions presented in the study are included in the article/Supplementary Materials, further inquiries can be directed to the corresponding authors.
