# Supplementary material for: Early Prediction of Tacrolimus-Induced Tubular Toxicity in Pediatric Refractory Nephrotic Syndrome Using Machine Learning
Source: Front Pharmacol. 2021 Aug 27;12:638724. doi: 10.3389/fphar.2021.638724 (PMC8430214; doi:10.3389/fphar.2021.638724)
Supplement: Supplementary file 1 [file DataSheet1.docx]

**Supplementary**

**TABLES**

**Table S1 The full name and abbreviation of the clinical variables included in this study.**

| **Abbreviation name** | **Full name** | **Abbreviation name** | **Full name** |
| --- | --- | --- | --- |
| AGE | Age at which toxicity occurs | PLT | Platelet count before toxicity |
| AGE0 | Age before administration | PLT0 | Platelet count before administration |
| ALB | Albumin levels before toxicity | PT | Pathological types |
| ALB0 | Albumin levels before administration | RP | Urine retinol-binding protein levels before toxicity |
| ALT | Alanine aminotransferase levels before toxicity | RP0 | Urine retinol-binding protein levels before administration |
| ALT0 | Alanine aminotransferase levels before administration | SCr | Serum creatinine levels before toxicity |
| ALTc | Changes in alanine aminotransferase levels | SCr0 | Serum creatinine levels before administration |
| AST | Aspartate aminotransferase levels before toxicity | SCrc | Changes in the serum creatinine levels |
| AST0 | Aspartate aminotransferase levels before administration | SDose_ADR_ | Total dose of steroid before toxicity |
| ASTc | Changes in aspartate aminotransferase levels | SFGR | Whether infection occurred before toxicity |
| Bβ2MG | Blood β2 microglobulin levels before toxicity | ST | Steroid types |
| Bβ2MG0 | Blood β2 microglobulin levels before administration | TC | Total cholesterol levels before toxicity |
| C3 | Blood complement 3 levels before toxicity | TC0 | Total cholesterol levels before administration |
| C3_0_ | Blood complement 3 levels before administration | TG | Triglyceride levels before toxicity |
| C4 | Blood complement 4 levels before toxicity | TG0 | Triglyceride levels before administration |
| C4_0_ | Blood complement 4 levels before administration | UACR | Urine microalbumin levels to urine creatinine levels ratio before toxicity |
| Comb-drugs-pd | Whether to combined use other immunosuppressants before toxicity | UACR0 | Urine microalbumin levels to urine creatinine levels ratio before administration |
| Comb-drugs-pk1 | Whether to combined use Wuzhi tablet, ketoconazole or fluconazole etc. when detecting tacrolimus concentration | UALB | Urine microalbumin levels before toxicity |
| Comb-drugs-pk2 | Whether to combined use rifampicin or Chinese herbal medicine when detecting tacrolimus concentration | UALB0 | Urine microalbumin levels before administration |
| Conc | Tacrolimus concentration | Ucr | Urine creatinine concentration before toxicity |
| CysC | Cystatin C levels before toxicity | UCr0 | Urine creatinine level before administration |
| CysC0 | Cystatin C levels before administration | UIgG | Urine immunoglobulin levels before toxicity |
| CysCc | Changes in cystatin C levels | UIgG0 | Urine immunoglobulin levels before administration |
| Daily Dose | Daily dose of tacrolimus at the first day | UPro | Urine protein level before toxicity |
| Daily Dose_ADR_ | Daily dose of tacrolimus before toxicity | UPro0 | Urine protein level before administration |
| DD | Blood D-Dimer levels before toxicity | UproQ | 24h urine protein levels before toxicity |
| DD0 | Blood D-Dimer levels before administration | UproQ0 | 24h urine protein levels before administration |
| D-duration | Disease duration before taking tacrolimus | Ur | Urea levels before toxicity |
| Dose_ADR_ | Total dose of tacrolimus before toxicity | Ur0 | Urea levels before administration |
| Fib | Fibrinogen levels before toxicity | URBC | Urine erythrocyte before toxicity |
| Fib0 | Fibrinogen levels before administration | URBC0 | Urine erythrocyte before administration |
| FSGS | Focal segmental glomurular sclerosis | US | Urine sugar level before toxicity |
| GENDER | Gender | US0 | Urine sugar level before administration |
| Glu | Blood sugar levels before toxicity | USG | Urine specific gravity before toxicity |
| Glu0 | Blood sugar level before administration | USG0 | Urine specific gravity before administration |
| HB | Hemoglobin level before toxicity | Uβ2MG | Urine β2 microglobulin levels before toxicity |
| HB0 | Hemoglobin level before administration | Uβ2MG0 | Urine β2 microglobulin levels before administration |
| HCT | Hematocrit before toxicity | Uβ2MGCR | Urine β2 microglobulin levels to urine creatinine levels ratio before toxicity |
| HCT0 | Hematocrit before administration | Uβ2MGCR0 | Urine β2 microglobulin levels to urine creatinine levels ratio before administration |
| K | Serum potassium level before toxicity | WBC | Leukocyte count before toxicity |
| K0 | Serum potassium level before administration | WBC0 | Leukocyte count before administration |
| MCD | Minimal change disease | WEIGHT | Weight before detecting concentration before toxicity |
| NAG | Urine N-acetyl β-D-glucosaminidase levels before toxicity | WEIGHT0 | Weight before administration |
| NAG0 | Urine N-acetyl β-D-glucosaminidase levels before administration | α1MG | Urine α1-microglobulin levels before toxicity |
| NAGc | Changes in urine N-acetyl β-D-glucosaminidase levels | α1MG0 | Urine α1-microglobulin levels before administration |

**Table S2 All single nucleotide polymorphisms (SNPs) included in this study.**

| **Coding genes of pharmacokinetic pathway-related proteins** | **Coding genes of transcriptional regulation related proteins** | **Coding genes of kidney disease related proteins** | **Coding genes of the other proteins** |
| --- | --- | --- | --- |
| *CYP3A4* rs2242480 | *CRTC2* rs8450 | *ACTN4* rs3745859 | *ADIPOQ* rs1501299 |
| *CYP3A4* rs4646437 | *DDIT3* rs1148557 | *ACTN4* rs56113315 | *ANGPTL4* rs1044250 |
| *CYP3A5*3* rs776746 | *DDIT3* rs697221 | *ACTN4* rs62121818 | *ANGPTL 4*rs2042899 |
| *CYP4F2* rs2108622 | *DDIT3* rs703835 | *ARHGAP24* rs17010887 | *ANGPTL4* rs4076317 |
| *MDR1* rs1045642 | *HNF4α* rs6031587 | *ARHGAP24* rs17010960 | *BAX* rs4645887 |
| *MDR1* rs1128503 | *IKBKB* rs9694985 | *ARHGDIA* rs11652950 | *BCL2* rs12457893 |
| *MDR1* rs2032582 | *JUN* rs4646999 | *ARHGDIA* rs34185514 | *BCL2* rs2850761 |
| *OATP1B1* rs2306283 | *JUN* rs4647001 | *CCN2* rs9399005 | *BCL2* rs34971240 |
| *POR* rs1057868 | *LMX1B* rs10733682 | *CD2AP* rs12664637 | *CCL2* rs1024611 |
| *POR* rs2868177 | *LMX1B* rs10987413 | *CD2AP* rs16876006 | *COQ2* rs10029891 |
|  | *LMX1B* rs10987417 | *CD2AP* rs4711880 | *COQ2* rs4693075 |
| **Coding genes of pharmacodynamic pathway-related proteins** | *LMX1B* rs13295990 | *CD2AP* rs719856 | *CTLA4* rs4553808 |
| *FKBP1A* rs1294689 | *LMX1B* rs2277158 | *CD2AP* rs9296560 | *HSPA5* rs12009 |
| *FKBP1A* rs1323135 | *LMX1B* rs71497630 | *INF2* rs1128880 | *IFNA1* rs1332190 |
| *FKBP5* rs1360780 | *LMX1B* rs7853174 | *INF2* rs12147772 | *IFNA1* rs1831583 |
| *FKBP5* rs9296158 | *NFKB1* rs230498 | *INF2* rs60468249 | *IFNA1* rs28383797 |
| *FKBP5* rs9470080 | *NFKB1* rs230526 | *ITGB4* rs2290460 | *KCNJ11* rs5219 |
| *MAP2K3* rs10468608 | *NFKB1* rs230532 | *ITGB4* rs62088212 | *LEP* rs2167270 |
| *MAP2K6* rs17223054 | *NFKB1* rs3774959 | *ITGB4* rs8669 | *MMP2* rs1132896 |
| *MAP2K6* rs17223089 | *NFKB1* rs4648050 | *ITGB4* rs871443 | *OLR1* rs1050283 |
| *MAP2K6* rs17223095 | *NFKBIA* rs8016947 | *ITGB4* rs9367 | *OLR1* rs1050286 |
| *MAP2K6* rs17690015 | *NR1H4* rs56163822 | *LAMB2* rs3768618 | *OLR1* rs10505755 |
| *MAP2K6* rs17823190 | *NR1I1* rs2853559 | *LAMB2* rs58189357 | *OLR1* rs11053646 |
| *MAP2K6* rs17823202 | *NR1I2* rs1951276 | *LAMB2* rs58325858 | *OLR1* rs34874375 |
| *MAP2K6* rs17823238 | *NR1I2* rs2233407 | *LAMB2* rs62119873 | *OLR1* rs3736232 |
| *MAP2K6* rs61289363 | *NR1I2* rs2276706 | *LAMB2* rs62119876 | *PPP2R3C* rs2415259 |
| *MAP3K11* rs1078457 | *NR1I2* rs3814055 | *LAMB2* rs62119877 | *PTPRO* rs11615050 |
| *MAP3K11* rs7946115 | *NR1I2* rs696 | *MYH9* rs12107 | *PTPRO* rs56260738 |
| *MAP3K11* rs948577 | *NR1I2* rs8904 | *MYH9* rs136206 | *ROS* rs17079069 |
| *MAP3K7* rs157702 | *PPARA* rs4253623 | *MYH9* rs2239781 | *ROS1*rs17079123 |
| *MAP3K7* rs157705 | *PPARA* rs4823613 | *MYH9* rs4821478 | *ROS1*rs529038 |
| *MAP3K7* rs205342 | *PPARA* rs5767743 | *MYH9* rs9619601 | *ROS1*rs2243380 |
| *MAP3K7* rs205343 | *RELA* rs2306365 | *MYO1E* rs56108450 | *SUMO4* rs237024 |
| *MAP3K7* rs791062 | *RELA* rs7119750 | *MYO1E* rs58948844 | *TGFB1* rs1800469 |
| *MAP3K7* rs967779 |  | *MYO1E* rs62004107 | *TGFB1* rs2073711 |
| *MAPK14* rs16884628 | **Coding genes of inflammatory cytokines** | *NPHS1* rs2071327 | *TGFB1* rs2241715 |
| *MAPK14* rs3804454 | *IL10* rs1800871 | *NPHS1* rs2285450 | *TGFB1*rs4803455 |
| *MAPK14* rs56156688 | *IL10* rs1800872 | *NPHS1* rs3814995 | *TGFB1* rs8105161 |
| *MAPK14* rs61763101 | *IL13* rs1295685 | *NPHS1* rs401824 | *TGFB2* rs61823428 |
| *MAPK14* rs851007 | *IL13* rs20541 | *NPHS1* rs437168 | *TGFB2* rs6683598 |
| *MAPK8* rs10776592 | *IL13* rs847 | *NPHS2* rs1079292 | *TGFB2* rs6704255 |
| *MAPK8* rs10857561 | *IL13* rs848 | *NPHS2* rs1410591 | *TGFB2* rs900 |
| *MAPK8* rs4838590 | *IL17* rs763780 | *NPHS2* rs1410592 | *TGFB2* rs991967 |
| *NFATC1* rs1660144 | *IL18* rs5744247 | *NPHS2* rs2274622 | *TGFB3* rs2268624 |
| *NFATC1* rs61658505 | *IL28B* rs12979860 | *NPHS2* rs2274623 | *TGFB3* rs2268626 |
| *NFATC1* rs754093 | *IL2RA* rs12722489 | *NPHS2* rs34693426 | *TGFB3* rs3917158 |
| *NFATC1* rs8090312 | *IL2RA* rs2104286 | *NPHS2* rs3818587 | *TGFB3* rs3917170 |
| *NFATC4* rs10141896 | *IL2RA* rs3118470 | *PDSS2* rs10214819 | *TLR4* rs1927907 |
| *PPP3CB* rs3763679 | *IL2RA* rs4147359 | *PDSS2* rs12200194 | *TLR4* rs1927914 |
| *PPP3CC* rs1075534 | *IL2RA* rs706778 | *PDSS2* rs17067991 | *TLR6* rs3775073 |
| *PPP3CC* rs2461483 | *IL2* rs2069762 | *PDSS2* rs8180652 | *TLR6* rs3821985 |
| *PPP3R1* rs1868402 | *IL2* rs2069763 | *PLCE1* rs11187870 | *TNFAIP3* rs3757173 |
| *PPP3R1* rs4347819 | *IL4R* rs1801275 | *PLCE1* rs17109671 | *ZMPSTE24* rs10489431 |
| *PPP3R1* rs4519508 | *IL4R* rs2107356 | *PLCE1* rs17417407 | *ZMPSTE24* rs2076697 |
|  | *IL4* rs2070874 | *PLCE1* rs2274223 | *ZMPSTE24* rs7516571 |
| **Coding genes of hormone invertase** | *IL4* rs2243250 | *PLCE1* rs2274224 |  |
| *HSD11B1* rs4844880 | *IL6R* rs2229238 | *PLCE1* rs35443777 |  |
| *HSD11B1* rs846908 | *TNF* rs1799724 | *SCARB2* rs61598131 |  |
| *HSD11B1* rs846910 | *TNF* rs1799964 | *SCARB2* rs6823680 |  |
|  | *TNF* rs1800610 | *SCARB2* rs7664889 |  |
|  |  | *SMARCAL1* rs11886806 |  |
|  |  | *SMARCAL1* rs184028 |  |
|  |  | *SMARCAL1* rs1983218 |  |
|  |  | *SMARCAL1* rs3755141 |  |
|  |  | *TRPC6* rs10501986 |  |
|  |  | *TRPC6* rs10895134 |  |
|  |  | *TRPC6* rs11224806 |  |
|  |  | *TRPC6* rs3824934 |  |
|  |  | *WT1* rs10742277 |  |
|  |  | *WT1* rs16754 |  |
|  |  | *WT1* rs1799925 |  |
|  |  | *WT1* rs1799937 |  |
|  |  | *WT1* rs5030207 |  |
|  |  | *WT1* rs5030317 |  |
|  |  | *WT1* rs5030320 |  |
|  |  | *WT1* rs9332974 |  |

**Table S3 The importance values of features screening by five models.**

| **Features** | **LR** | **XGBoost** | **ET** | **RF** | **GBDT** |
| --- | --- | --- | --- | --- | --- |
| *TRPC6*rs3824934_GG | -1.514 | 0.241 | 0.060 | 0.263 | 0.225 |
| *HSD11B1*rs846910_AG | -1.638 | 0.132 | 0.411 | 0.405 | 0.270 |
| *SCARB2*rs6823680_CC | 0.501 | 0.276 | 0.307 | 0.119 | 0.206 |
| *MAP2K6*rs17823202_GG | -0.970 | 0.350 | 0.223 | 0.213 | 0.299 |
